# Adaptive Strategies for the Elderly in Inhibiting Irrelevant and Conflict No-Go Trials while Performing the Go/No-Go Task

**DOI:** 10.3389/fnagi.2015.00243

**Published:** 2016-01-06

**Authors:** Shulan Hsieh, Mengyao Wu, Chien-Hui Tang

**Affiliations:** ^1^Cognitive Electrophysiology Laboratory: Control, Aging, Sleep, and Emotion (CASE), Department of Psychology, National Cheng Kung UniversityTainan, Taiwan; ^2^Institute of Allied Health Sciences, National Cheng Kung UniversityTainan, Taiwan; ^3^Department of Occupational Therapy, Shu-Zen Junior College of Medicine and ManagementKaohsiung, Taiwan

**Keywords:** event-related potential, distraction, inhibition, no-go, probability

## Abstract

This study aimed to differentiate whether or not older adults are more prone to distraction or conflict, as induced by irrelevant and conflict no-go stimuli (irNOGO and cfNOGO), respectively. This study also aimed to determine whether or not older adults would devote more effort to withholding a no-go trial in the higher-control demand condition (20% no-go trials’ probability) as compared to the lower-control demand condition (50 and 80% no-go trials’ probability). A total of 96 individuals were recruited, and each of the three no-go trials’ probability conditions included 32 participants (16 younger adults and 16 older adults). Both behavioral and event-related potential (ERP) data were measured. The behavioral results showed that the older adults performed more poorly than the younger adults for the go trials, as reflected by slower reaction times (RTs) and higher numbers of omission errors in the go trials. In contrast, in the no-go trials, the older adults counter-intuitively exhibited similar behavioral performance (i.e., equivalent commission errors) as compared to the younger adults. The ERP data further showed that the older adults (but not the younger adults) exhibited larger P3 peak amplitudes for the irNOGO than cfNOGO trials. Yet, on the other hand, the older adults performed more poorly (i.e., had more commission errors) in the cfNOGO than irNOGO trials. These results seem to suggest that the older adults recruited more control processes in order to conquer the commitment of responses in the no-go trials, especially in the irNOGO trials. This age-related compensatory response of recruiting more control processes was specifically seen in the 20% no-go trial probability condition. This study therefore provides a deeper understanding into how older adults adopt strategies for performing the go/no-go task such as devoting more control processes to inhibiting the irNOGO trials compared to the cfNOGO trials in order to cope with their deficient inhibition ability.

## Introduction

Daily life often requires an individual to produce a certain behavior in an environment that is filled with irrelevant and often distracting information. Hence, to achieve the goal of acting adequately, one has to adaptively overcome the competition brought by the strong, yet inappropriate, momentary tendency to inhibit a prepotent yet unintentional response in order to prevent negative consequences. These adaptive processes are broadly termed *inhibition* or *suppression*, and they have often been hypothesized to involve the frontal lobe functions (e.g., Rogers et al., 1998). Given the importance of inhibition in everyday life, several studies have attempted to address the issue of whether or not elderly people could successfully cope with brain degeneration, particularly in the frontal lobes (Dempster, 1992; Raz, 2000; West, 2000; Dennis and Cabeza, 2008), to achieve the goal of acting adequately in everyday life scenarios.

However, the findings regarding how capable an elderly person can be in performing an inhibition-related task are equivocal: some studies showed that the elderly suffered from generic inhibition deficit, hence, they were incapable of performing any inhibition-related task as compared to the younger adults (Hasher and Zacks, 1988; Bokura et al., 2002; Vallesi et al., 2009; Vallesi, 2011; Lucci et al., 2013; Pires et al., 2014), while some other studies have shown that the elderly could develop some strategies to compensate for their deficiency in achieving the task goal (Cabeza et al., 2002; Phillips and Andrés, 2010; Hsieh and Fang, 2012; Hsieh et al., 2012; Hsieh and Lin, 2014). Many factors such as different experimental settings, different populations, and different types of stimuli and requirements could potentially contribute to the discrepancies. Among these factors, we suggest that task parameters may play a critical role in older adults’ performance strategies. Hence, in this study we manipulated two task parameters in a go/no-go task paradigm to address the inhibition proficiency of older adults. We focused specifically on a go/no-go task paradigm because such an inhibition task has been proven to be sensitive to aging (Bokura et al., 2002; Vallesi et al., 2009; Vallesi, 2011; Lucci et al., 2013; Pires et al., 2014). The two-task parameters we manipulated here include a no-go stimulus and a no-go stimulus probability. With regard to the type of no-go stimulus, we incorporated two types of no-go stimuli: irrelevant no-go (irNOGO) and conflict no-go (cfNOGO). The irNOGO stimuli belong to a different semantic category from that of the go stimuli (e.g., numbers vs. letters), which yields an obvious distinction between the target (go) and non-target (no-go) stimuli, even though the no-go stimuli may share a common feature with the go stimuli. For example, both the irNOGO and go stimuli could be either red or blue, but they belong to numbers and letters, respectively. In contrast, the cfNOGO stimuli share the same category (i.e., letters) as the go stimuli. The rationale for this manipulation is that the irNOGO stimuli should be easier to distinguish from the go stimuli; therefore, it is easier inhibit compared to the cfNOGO stimuli. On the other hand, some prior studies in the memory research domain have suggested that older adults were more vulnerable to internal distraction and weakening concentration skills due to their subtle changes in brain activity (e.g., Grady et al., 2006). It is therefore interesting to examine whether or not older adults are also susceptible to external distractions, such as those induced by the irNOGO stimuli in a go/no-go task.

With regard to no-go stimulus probability, we manipulated the probability of no-go trials in a block to be either 20, 50, or 80%. The rationale for the probability manipulation is that with a strong response bias towards go stimuli (20% no-go probability), one can maximize the engagement of executive control to inhibit no-go stimuli (Bruin and Wijers, 2002; Ford et al., 2004). Subsequently, the performance of a go/no-go task may be more sensitive to aging in the condition of this higher demand condition (20% of no-go probability).

With the manipulation of these two task parameters, we could examine whether or not aging results in a selective deficit in which task parameters are sensitive to the effect of aging in only some scenarios. Because no overt response can be recorded for no-go trials in a go/no-go paradigm, a neural imaging approach is needed to uncover the underlying processes of the no-go trials. In this study, we employed the event-related potential (ERP) method, which yields higher temporal resolution and uncovers the underlying neural activity for no-go trials.

**Table 1:** **The demographic information for all participants in all conditions**.

	20% no-go		50% no-go		80% no-go	
	Younger	Older	Younger	Older	Younger	Older
Gender (male/female)	7/9	7/9	8/8	8/8	7/9	7/9
Age (year)	21.31 (1.36)	66.38 (4.43)	21.31 (1.26)	67.25 (4.59)	21.63 (1.49)	68.63 (5.88)
Education (year)	15.69 (1.21)	12.69 (2.08)	15.00 (1.06)	12.56 (3.48)	15.44 (1.17)	13.31 (2.66)
BDI	3.44 (2.06)	5.25 (4.71)	4.69 (3.58)	6.69 (3.58)	3.69 (2.17)	4.25 (3.44)
MMSE	28.63 (0.60)	27.06 (1.14)	28.50 (0.71)	26.69 (1.10)	28.19 (1.01)	27.19 (0.73)

*BDI, Beck Depression Inventory; MMSE, Mini-Mental State Examination; the value in each cell denotes the mean, and the value in each bracket denotes standard deviation*.

Two ERP components are considered in this study that have been demonstrated to be sensitive to a go/no-go task, that is, the stimulus-locked P3 and N2 components (for a review, see Pires et al., 2014). Stimuli that trigger a tendency to make incorrect prepotent responses (e.g., incongruent or no-go stimuli) have been found to be associated with enhanced fronto-central N2 amplitude, which is thought to reflect inhibition (Van Boxtel et al., 2001; Roche et al., 2005) and/or response conflict control processes (Falkenstein et al., 2001; Van Veen and Carter, 2002a,b; Nieuwenhuis et al., 2003). Furthermore, Nieuwenhuis et al. (2003) observed that no-go N2 amplitudes were higher in the high go-prepotency (20% no-go) condition compared with medium (50%) and low go-prepotency (80% no-go) conditions, which supports the conflict theory of N2. Following the N2, there is a positive ERP component that peaks at approximately 250–500 ms for both go (known as the go P3) and no-go (known as the no-go P3) trials. Both go and no-go P3s may reflect context updating (Donchin and Coles, 1988), which is necessary for successful ongoing execution and inhibition of prepotency responses. Specifically, the no-go P3 has been shown to be closely related to inhibition (Roberts et al., 1994; Fallgatter and Strik, 1999; Tekok-Kilic et al., 2001; Smith et al., 2008). Hence, by observing how aging may modulate the no-go N2 and P3 components, we can uncover age-related inhibition and/or conflict processes.

## Materials and Methods

### Participants

A total of 96 individuals were recruited through the internet and local community advertisements; each of the three no-go probability condition blocks included 32 participants (16 younger adults and 16 older adults). For the 20% no-go probability condition, 16 young adults (9 females) had a mean age of 21.31 ± 1.40 years (range 20–25 years) and an average of 15.69 ± 1.25 years of education; the 16 elderly adults (9 females) had a mean age of 66.38 ± 4.57 years (range 61–72 years) and an average of 12.69 ± 2.15 years of education. For the 50% no-go probability condition, the 16 young adults (8 females) had a mean age of 21.31 ± 1.30 years (range 20–25 years) and an average of 15.00 ± 1.10 years of education; the 16 elderly adults (8 females) had a mean age of 67.25 ± 4.74 years (range 60–76 years) and an average of 12.56 ± 3.60 years of education. For the 80% no-go probability condition, the 16 young adults (9 females) had a mean age of 21.63 ± 1.54 years (range 19–24 years) and an average of 15.44 ± 1.21 years of education; the 16 elderly adults (9 females) had a mean age of 68.63 ± 6.08 years (range 61–80 years) and an average of 13.31 ± 2.75 years of education (see Table 1).

The 2-way analysis of variance (ANOVAs) on age with two between-subjects factors of age and no-go probability showed a significant main effect of age (young: 21.42 ± 1.38 years vs. old: 67.42 ± 5.09 years, *F*_(1,90)_ = 3529.05, *p* < 0.01), but it did not show a significant main effect of no-go probability or a significant interaction between age and no-go probability (i.e., all *ps* > 0.05). The 2-way ANOVA on years of education showed a significant effect of age (young: 12.85 ± 2.82 years vs. old: 15.38 ± 1.18 years, *F*_(1,90)_ = 31.18, *p* < 0.01). No significant effect of no-go probability or interaction between age and no-go probability was found (all *ps* > 0.05).

All participants provided their written informed consent, and the study protocol was approved by the Institutional Review Board (IRB) of the National Cheng Kung University Hospital, Taiwan. All participants were paid NT $500–1000 (US $15–30) for approximately 3 h of participation. All participants were right-handed, free of neurological and psychological disorders, and had normal or corrected-to-normal vision. The Mini-Mental State Examination (MMSE; Folstein et al., 1975) screened all participants for dementia based on the following screening criteria: 25–30 points = normal; 21–24 points = mild dementia; 14–20 points = moderate dementia; and ≤13 points = severe dementia. For the 20% no-go probability condition, the mean MMSE score was 28.63 ± 0.60 for the younger adults and 27.06 ± 1.14 for the older adults. For the 50% no-go probability condition, the mean MMSE score was 28.50 ± 0.71 for the younger adults and 26.68 ± 1.10 for the older adults. For the 80% no-go probability condition, the mean MMSE score was 28.18 ± 1.01 for the younger adults and 27.19 ± 0.73 for the older adults.

The 2-way ANOVA on the MMSE score showed a significant effect of age (young: 26.98 ± 1.03 vs. old: 28.44 ± 0.81, *F*_(1,90)_ = 58.15, *p* < 0.01) but no significant effect of no-go probability; also, there was no significant interaction between age and no-go probability (all *ps* > 0.05). Since there was a significant effect of age on MMSE scores, this may be a potential confounding variable for the results, i.e., the changes may be due to cognitive decline rather than physiological aging. In order to preclude the possible contribution of cognitive decline in the older group, we have additionally run an analysis of covariance (ANCOVA) using MMSE as a covariate factor. The results of the ANCOVA showed the same patterns as those of the ANOVA reported here. Hence, although the older adults exhibited lower MMSE scores, they did not confound the current findings.

### Stimuli

The stimuli were generated using E-Prime software (Psychology Software Tools, Inc., Pittsburgh, PA, USA) and were presented in red or blue against a black background on the center of a computer screen that was placed at a distance of 90 cm from the participant. In this task, go stimuli were red or blue vowels (“A”, “E”, “I”, “U”) and no-go stimuli were either red or blue consonants (“L”, “N”, “P”, “Z”; designed as cfNOGO stimuli) or red and blue numbers (“3”, “4”, “5”, “6”; designed as irNOGO stimuli).

### Design and Procedure

**Figure 1:**
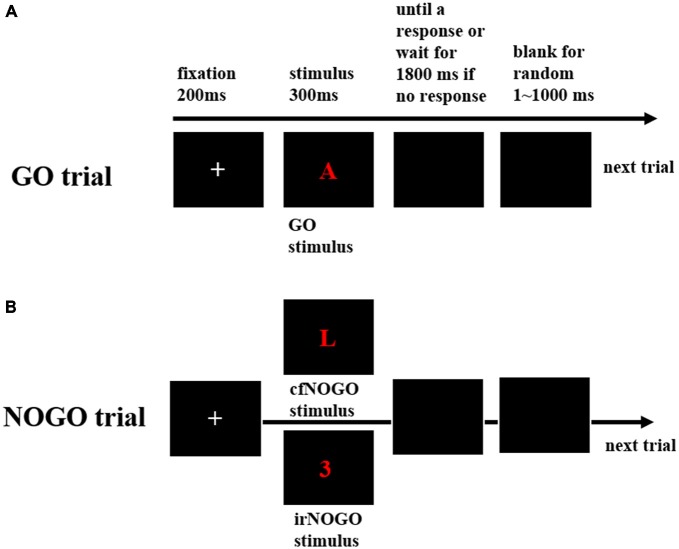
**The behavioral paradigm. (A)** Schematic representation of the events in a go trial, including a fixation, followed by a go stimulus; **(B)** Schematic representation of the events in a no-go trial, including a fixation, followed by either of the two no-go stimuli, irrelevant (irNOGO) and conflict (cfNOGO).

Each trial began with the presentation of a white fixation cross “+” for a duration of 200 ms, followed by a go or no-go stimulus for a duration of 300 ms. This was then replaced by a black blank screen that awaited a response or until 1800 ms elapsed if no response was recorded. Also, there was an additional waiting duration that varied randomly between 1–1000 ms before the next trial commenced (see Figure 1). Participants were required to make (for a go stimulus) or withhold (for a no-go stimulus) a response as soon and as accurately as possible.

The total number of trials in the no-go probability condition block was 960. The probabilities of no-go stimuli were 20% (192 trials), 50% (480 trials), and 80% (768 trials), respectively. Among the no-go trials, cfNOGO and irNOGO stimuli comprised 50% each. There were at least 36 practice trials (more trials if necessary) before each condition block. During the practice session, feedback of “correct” or “incorrect” was given at the end of each trial to facilitate the participants’ familiarization with the response rule.

### EEG Recording

The participants were seated in a comfortable chair in a sound-attenuated room during the experiment. Electroencephalography (EEG) activity was continuously recorded using Neuroscan SynAmp2 amplifier and Q-Cap (Neuroscan Q-Cap: AgCl-32 electrode cap; Neuroscan, Inc., El Paso, TX, USA) from 32 scalp electrodes. The vertical electrooculogram (EOG) was recorded by two electrodes 2 cm above and 2 cm below the left eye, and the horizontal EOG was recorded by two electrodes 1 cm external to the outer canthus of each eye. A ground electrode was placed on the forehead. The electrodes were initially referenced online to the left mastoid and offline to the average of the left and right mastoids. Electrode impedances were maintained below 5 kΩ. The EEG and EOG signals were amplified and digitized at a sampling rate of 500 Hz, with an online high-pass filter of 0.1 Hz and a low-pass filter of 30 Hz. Ocular artifacts associated with blinks were corrected by the ocular reduction command offered by the Neuroscan software (Neuroscan, Inc., El Paso, TX, USA) and were then further removed via an algorithm (Neuroscan software) that rejected any epoch if the signal was below −50 ± 50 μV, if the drift of the EEG from baseline exceeded −50 ± 50 μV, or if the A/D converts became saturated. The total rejection rate across the various conditions averaged approximately 23, 28 and 24% for each no-go probability condition block, respectively.

### Event-Related Potential (ERP) Analysis

We focused on the N2 and P3 components as they have been hypothesized to link inhibition and response conflict control processes. Moreover, because go and no-go trials involve different processes with respect to motor execution, we separated the ERP analyses of go trials from those of no-go trials. In each condition block, stimulus-locked epochs were taken from the continuous EEG signal and time-locked to the onset of the go/no-go stimulus from −50 ms to 800 ms for all recording channels. For each channel, all stimulus-locked epochs were baseline-corrected by obtaining the mean level of activity in the period from 50 ms before to 50 ms after target onset and then subtracting that average from the level of activity at the sample point. The N2 was found to be maximal at the FCz site, whereas the P3 was maximal at the Cz site in this study. Hence, we searched for the peak-to-peak amplitude at the FCz site during the time windows of 150–350 ms (positive dip) and 250–450 ms (negative peak) following the onset of the stimulus and then computed the voltage difference between the positive dip and negative peak as the stimulus-locked N2 peak-to-peak amplitude. Then, we searched for the peak latency and peak amplitude at the Cz site during the time window of 300–750 ms following the onset of the stimulus and then computed the voltage difference between the peak amplitude relative to the baseline as the stimulus-locked P3 peak amplitude and the time point at which the P3 peak amplitude occurred as the P3 peak latency.

### Statistical Analysis

The 2-way ANOVAs for the go trials were performed on the behavioral and ERP data, respectively, with two between-subject factors of age and no-go probability. The 3-way ANOVAs for the no-go trials were performed on the behavioral and ERP data, respectively, with two between-subjects factors of age and no-go probability and one within-subject factor of no-go stimulus types. *Post hoc* analysis following the significant effect of no-go probability (with three levels) was performed using Tukey tests. When two (or more) factors in the ANOVA showed a statistically significant interaction, we carried on analyzing the simple main effects which involve the examination of the effect of one factor at one level of the other factor. That is, the data were split for each level of one factor and one-way ANOVAs were conducted. Like any other one-way ANOVA with more than two levels, after the significant F, a *post hoc* Tukey test was conducted to find out which pair (or pairs) of means was (were) statistically different. To overcome the inflation of Type 1 error when a series of simple main effect analyses were conducted, we used the Bonferroni correction to adjust the *p* value. In addition, in the choice of error term for simple main effect test, we followed the pooled error term approach advised by Howell (2010). That is, Mean Square Error (MSE) from the original factorial ANOVA was used as opposed to MSE from the follow-up one-way ANOVA in calculating the F for the simple main effects. The detailed steps for the calculation are described in Howell’s (2010) book (e.g., ps. 483–488 in his book, “*Statistical Methods for Psychology*”).

## Results

### Behavioral Data Analysis

**Figure 2:**
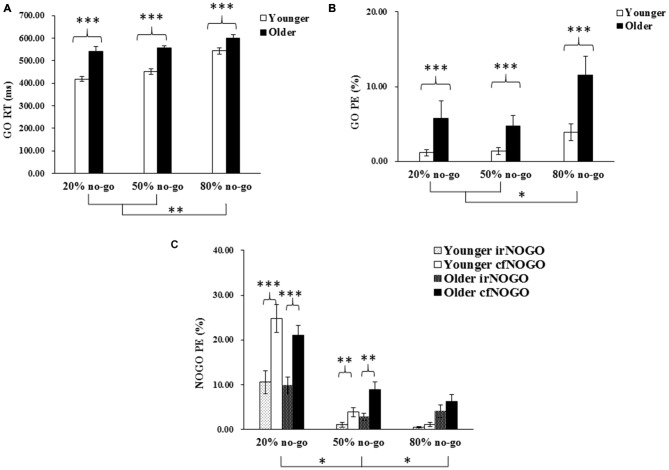
**(A)** Mean response times (RTs; and standard errors of the mean) of correct responses (in milliseconds) to go stimuli as a function of age and no-go probability condition; **(B)** Mean percentage of errors (PE; and standard errors of the mean) of omitted responses to go stimuli as a function of age and no-go probability condition; **(C)** Mean percentage of errors (PE; and standard errors of the mean) of committed responses to no-go stimuli as a function of age and no-go probability condition. Significant effects are highlighted with *p < 0.05, **p < 0.01, or ***p < 0.001.

The first trial of each block and trials with RTs faster than 150 ms or slower than 1500 ms were discarded from further analysis. The behavioral results are shown in Figure 2, including the go trials’ RT (Figure 2A) and omission rates (Figure 2B) and the no-go trials’ commission errors (Figure 2C).

### Reaction Time (RT) on Go Trials

The 2-way ANOVA on the RTs of the go trials showed that the younger adults generally performed faster than the older adults, *F*_(1,90)_ = 63.11, *p* < 0.001, and that RTs were faster in the 20% no-go probability (high go-prepotency) and the 50% no-go probability (medium go-prepotency) conditions than in the 80% no-go probability (low go-prepotency) condition (no-go probability, *F*_(2,90)_ = 21.42, *p* < 0.001; all Tukey tests: *ps* < 0.005). There was no significant interaction between the two factors, *F*_(2,90)_ = 2.61, *p* = 0.08.

### Percentage of Error (PE) on Go Trials: Omission Errors

The 2-way ANOVA on the omission errors of the go trials showed that the younger adults generally performed better (fewer omission errors) than the older adults, *F*_(1,90)_ = 15.20, *p* < 0.001, and that omission errors were fewer in the 20 and 50% no-go probability conditions (3.46 and 3.04%) than in the 80% no-go probability condition (7.70%; *F*_(2,90)_ = 4.92, *p* < 0.01; all Tukey tests: *p* < 0.05). There was no significant interaction between the two factors, *F*_(2,90)_ = 0.93, *p* = 0.40.

### Percentage of Error (PE) on No-Go Trials: Commission Errors

The 3-way ANOVA on the commission errors of the no-go trials showed that there was no significant effect of age, *F*_(1,90)_ = 2.14, *p* = 0.15, but there was a significant effect of no-go probability, *F*_(2,90)_ = 47.41, *p* < 0.0001. Commission errors were more frequent in the 20% no-go probability condition (16.53%) than in the 50% (4.13%) and 80% no-go probability conditions (2.95%; all Tukey tests: *ps* < 0.05). There was also a significant effect of no-go stimulus type, *F*_(1,90)_ = 88.31, *p* < 0.0001, showing that irNOGO (4.78%) stimuli elicited fewer commission errors than cfNOGO (10.96%) stimuli. There was also a significant 2-way interaction between no-go probability and no-go stimulus type, *F*_(2,90)_ = 26.52, *p* < 0.0001. Follow-up tests for this interaction showed that the effect of no-go probability was significant on both cfNOGO trials (*F*_(2,90)_ = 71.51, *p* < 0.0001) and irNOGO trials (*F*_(2,90)_ = 14.41, *p* < 0.001), yet there was a larger effect on the cfNOGO trial type. In addition, the simple effect of the no-go stimulus type (fewer commission errors for the irNOGO than cfNOGO trials) was significant only in the 20 and 50% no-go probability conditions.

### Summary of Behavioral Data

To summarize, the older adults appeared to perform more poorly than the younger adults on go trials, as reflected by their slower go RTs and higher go omission errors; this age effect was not modulated by no-go probability. In contrast, the older adults counter-intuitively exhibited similar behavioral performance (i.e., equivalent commission errors) as compared to the younger adults. In addition, it appeared to be more difficult for both age groups to withhold a response to cfNOGO stimuli compared to irNOGO stimuli, based on the data of commission errors.

### Event-Related Potential (ERP) Data

**Figure 3:**
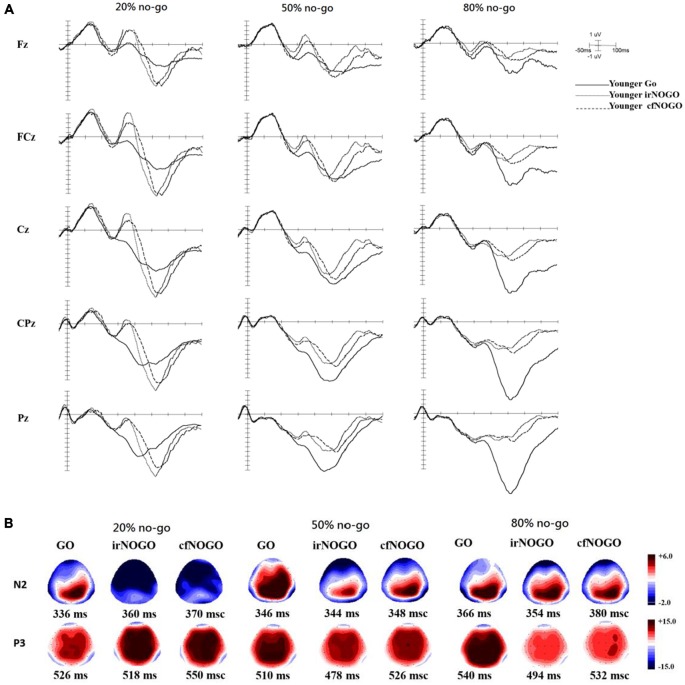
**(A)** ERP waveforms as a function of no/no-go condition (GO, irNOGO, cfNOGO) and no-go probability condition at five representative electrodes of Fz, FCz, Cz, CPz, and Pz for the younger adults. **(B)** Topographic maps of the GO N2, irNOGO N2, cfNOGO N2, GO P3, irNOGO P3, and cfNOGO P3, separately for each no-go probability condition and for the younger adults. Each map describes the topographic distribution at the peak latency for each condition.

**Figure 4:**
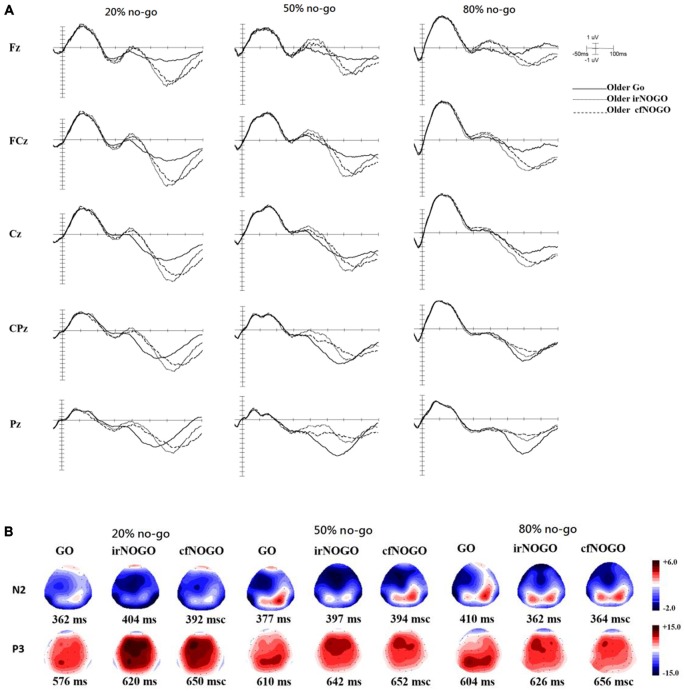
**(A)** ERP waveforms as a function of no/no-go condition (GO, irNOGO, cfNOGO) and no-go probability condition at five representative electrodes of Fz, FCz, Cz, CPz, and Pz for the older adults. **(B)** Topographic maps of the GO N2, irNOGO N2, cfNOGO N2, GO P3, irNOGO P3, and cfNOGO P3, separately for each no-go probability condition and for the older adults. Each map describes the topographic distribution at the peak latency for each condition.

ERPs associated with each condition and age group at the Fz, FCz, Cz, CPz, and Pz sites as well as topographic maps are shown in Figures 3A,B for go trials and Figures 4A,B for no-go trials.

### P3 Peak Latency on Go Trials

**Figure 5:**
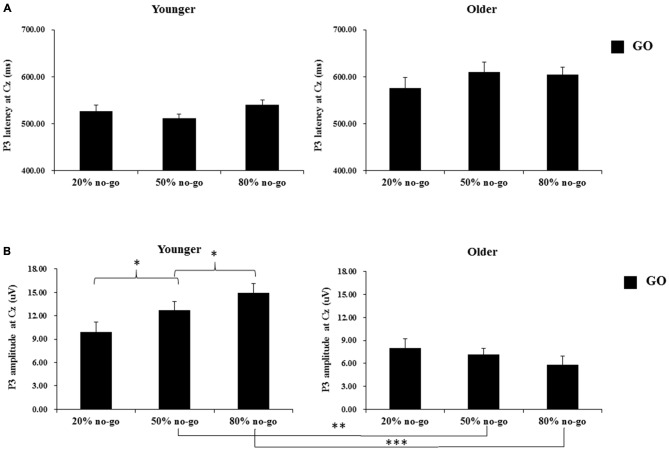
**(A)** Grand mean P3 peak latencies (in milliseconds) at the Cz site as a function of no-go probability condition for GO trials and for the younger (upper left panel) and older (upper right panel) adults, respectively. Error bars represent standard errors; **(B)** Grand mean P3 peak amplitudes (in μV) at the Cz site as a function of no-go probability condition for GO trials and for the younger (lower left panel) and older (lower right panel) adults, respectively. Error bars represent standard errors. Significant effects are highlighted with *p < 0.05, **p < 0.01, or ***p < 0.001.

The 2-way ANOVA on the P3 peak latencies of go trials showed that the younger adults exhibited earlier P3 peak latencies than the older adults (young: 526.04 ms vs. old: 596.75 ms), *F*_(1,90)_ = 26.63, *p* < 0.0001, but there was no significant effect of no-go probability, *F*_(2,90)_ = 0.81, *p* = 0.45. There was also no significant 2-way interaction between age and no-go probability, *F*_(2,90)_ = 1.11, *p* = 0.34 (see Figure 5A).

### P3 Peak Amplitude on Go Trials

The 2-way ANOVA on the P3 peak amplitudes of the go trials showed larger P3 peak amplitudes for the younger (12.53 μV) than older (7.01 μV) adults, *F*_(1,90)_ = 34.58, *p* < 0.0001, but no significant main effect of no-go probability, *F*_(2,90)_ = 0.79, *p* = 0.46. Yet, there was a significant 2-way interaction between age and no-go probability, *F*_(2,90)_ = 4.94, *p* < 0.01. A simple effect test following this interaction showed that the significant main effect of no-go probability occurred only for the younger adults, *F*_(2,90)_ = 4.80, *p* < 0.05, but not for the older adults, *F*_(2,90)_ = 0.93, *p* = 0.40, and that the significant effect of age occurred in the 50%, *F*_(1,90)_ = 11.49, *p* < 0.005, and 80%, *F*_(1,90)_ = 31.59, *p* < 0.0001, no-go probability conditions but not in the 20% no-go probability conditions, *F*_(1,90)_ = 1.38, *p* = 0.24 (see Figure 5B).

### P3 Peak Latency on No-Go Trials

**Figure 6:**
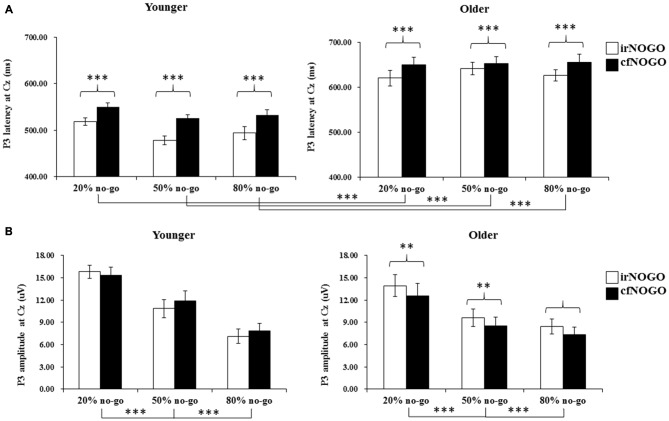
**(A)** Grand mean P3 peak latencies (in milliseconds) at the Cz site as a function of no-go probability condition for NOGO (irNOGO and cfNOGO) trials and for the younger (upper left panel) and older (upper right panel) adults, respectively. Error bars represent standard errors; **(B)** Grand mean P3 peak amplitudes (in μV) at the Cz site as a function of no-go probability condition for NOGO (irNOGO and cfNOGO) trials and for the younger (lower left panel) and older (lower right panel) adults, respectively. Error bars represent standard errors. Significant effects are highlighted with **p < 0.01, or ***p < 0.001.

The 3-way ANOVA on the P3 peak latencies of no-go trials showed that the younger adults exhibited earlier P3 peak latencies than the older adults (young: 516.35 ms vs. old: 641.46 ms), *F*_(1,90)_ = 162.85, *p* < 0.001, and that irNOGO stimuli elicited earlier P3 peak latencies than cfNOGO stimuli, *F*_(1,90)_ = 30.42, *p* < 0.0001. There were no significant effect of no-go probability and any interactions (age × type of no-go stimulus: *F*_(1,90)_ = 2.03, *p* = 0.16; no-go probability × type of no-go stimulus: *F*_(2,90)_ = 0.08, *p* = 0.93; age × type of no-go stimulus × no-go probability: *F*_(2,90)_ = 0.922, *p* = 0.40, see Figure 6A.

### P3 Peak Amplitude on No-Go Trials

The 3-way ANOVA on the P3 peak amplitudes of the no-go stimuli showed that P3 peak amplitudes for the no-go trials were larger in the 20% no-go probability condition than in the 50 and 80% no-go probability conditions, *F*_(2,90)_ = 15.58, *p* < 0.0001 (all Tukey tests: *ps* < 0.01). There was also a significant 2-way interaction between age and no-go stimulus type, *F*_(1,90)_ = 13.49, *p* < 0.001.

A simple effect test on the interaction between age and no-go stimulus type showed that only older adults exhibited larger P3 peak amplitudes for irNOGO stimuli than for cfNOGO stimuli but not for the younger adults (young: *F*_(1,90)_ = 1.80, *p* = 0.19; old: *F*_(1,90)_ = 14.83, *p* < 0.001; see Figure 6B).

### Summary of the P3 Findings

#### Go P3 Peak Latency and Amplitude

For the go P3 peak latency, there was a significant effect of age that showed an earlier P3 peak latency for the younger adults. As for the go P3 peak amplitude, the significant effect of aging (younger larger than older) occurred only in the 50 and 80% no-go probability conditions. In addition, the significant effect of no-go probability occurred only for the younger adults, i.e., showing smaller go P3 peak amplitudes in the 20% than 80% no-go probability condition but not for the older adults.

#### No-Go P3 Peak Latency and Amplitude

The significant effect of age was found for the overall no-go P3 peak latency. In addition, both age groups exhibited earlier P3 peak latencies for the irNOGO trials compared to the cfNOGO trials. As for the no-go P3 peak amplitude, there was a significant effect of no-go probability for both age groups, showing larger no-go P3 peak amplitudes in the 20% no-go probability conditions compared to the 50 and 80% no-go probability conditions. More importantly, only the older adults exhibited larger P3 peak amplitudes for the irNOGO trials compared to the cfNOGO trials. These results suggested that while the younger adults exhibited similar no-go P3 peak amplitudes between the irNOGO and cfNOGO trials, the older adults exhibited more prominent P3 peak amplitudes for the irNOGO than cfNOGO trials. Subsequently, this phenomenon resulted in a significant effect of age for the cfNOGO trials but not for the irNOGO trials.

### N2 Peak-to-Peak Amplitude on Go Trials

**Figure 7:**
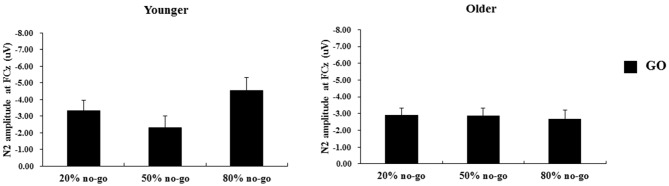
**Grand mean N2 peak-to-peak amplitudes (in μV) at the FCz site as a function of no-go probability condition for GO trials and for the younger (left panel) and older (right panel) adults, respectively. Error bars represent standard errors**.

The 2-way ANOVA on the N2 peak-to-peak amplitudes of the go trials showed no significant effects or interactions (age: *F*_(1,90)_ = 1.24, *p* = 0.27; no-go probability: *F*_(2,90)_ = 1.35, *p* = 0.27; age *x* no-go probability: *F*_(2,90)_ = 1.89, *p* =0.19; see Figure 7).

### N2 Peak-to-Peak Amplitude on No-Go Trials

The 3-way ANOVA on the N2 peak-to-peak amplitudes of no-go stimuli showed that the younger adults exhibited larger N2 peak-to-peak amplitudes (−5.97 μV) than the older adults (−4.52 μV), *F*_(1,90)_ = 8.24, *p* < 0.01. Also, N2 peak-to-peak amplitudes were larger in the 20% no-go probability condition compared to the 50% no-go probability condition and amplitudes in the latter condition were larger than amplitudes in the 80% no-go probability condition, *F*_(2,90)_ = 33.87, *p* < 0.0001 (all Tukey tests: *ps* < 0.01). There was also a significant effect of no-go stimulus type, *F*_(1,90)_ = 7.61, *p* < 0.01; this indicates that irNOGO stimuli elicited larger N2 peak-to-peak amplitudes than cfNOGO stimuli. There were significant 2-way interactions between age and no-go probability, *F*_(2,90)_ = 3.78, *p* < 0.05, and between no-go probability and no-go stimulus type, *F*_(2,90)_ = 7.34, *p* < 0.005.

The simple effect test for the interaction between age and no-go probability showed that the effect of age was only significant for the 20% no-go probability condition, where the younger adults exhibited larger N2 peak-to-peak amplitudes than older adults. On the other hand, both age groups exhibited significant effects for the no-go probability on the N2 peak-to-peak amplitude.

**Figure 8:**
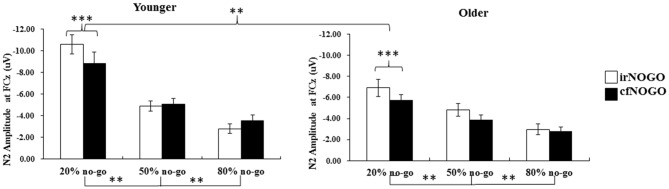
**Grand mean N2 peak-to-peak amplitudes (in μV) at the FCz site as a function of no-go probability condition for NOGO (irNOGO and cfNOGO) trials and for the younger (left panel) and older (right panel) adults, respectively.** Error bars represent standard errors. Significant effects are highlighted with **p < 0.01, or ***p < 0.001.

The simple effect test on the interaction of no-go probability and no-go stimulus type nevertheless showed a significant main effect of no-go stimulus type (i.e., the irNOGO trials elicited larger N2 amplitudes than cfNOGO trials) in the 20% no-go probability condition, *F*_(1,90)_ = 20.03, *p* < 0.0001, but not for the 50 and 80% no-go probability condition (50%: *F*_(1,90)_ = 1.46, *p* = 0.23; 80%: *F*_(1,90)_ = 0.81, *p* = 0.37). Furthermore, for both the irNOGO and cfNOGO stimuli, the amplitudes were larger in the 20% no-go probability condition than in the 50 and 80% no-go probability condition (all *ps* < 0.05; see Figure 8).

### Summary of the N2 Findings

#### Go N2 Peak-to-Peak Amplitude

The significant effect of no-go probability on go N2s occurred only for the younger adults and between the 50 and 80% no-go probability conditions, whereas none of the significant main effects and interactions were found for the older adults.

#### No-Go N2 Peak-to-Peak Amplitude

The significant effect of age (i.e., the younger adults exhibited larger N2 amplitudes than the older adults) on no-go N2s occurred mainly in the 20% no-go probability condition. In addition, the significant effect of no-go trial type (i.e., irNOGO trials exhibited larger N2 amplitudes than cfNOGO trials) was found only for the 20% no-go probability condition for both age groups. Furthermore, for both the irNOGO and cfNOGO stimuli, the amplitudes were larger in the 20% no-go probability condition than in the 50 and 80% no-go probability conditions.

## Discussions

The aim of this study was to evaluate whether older adults exhibited selective inhibition deficit by using a go/no-go paradigm with the manipulation of no-go stimulus-type and no-go probability. The current behavioral results showed that the older adults performed more poorly than the younger adults as reflected from their slower RTs and their higher omission errors in the go trials. This age difference was not further modulated by no-go probability, despite the fact that there was a significant effect of no-go probability on both RTs and omission errors. Interestingly, in contrast to the inferior performance on go trials for the older adults compared to the younger adults, the older adults counter-intuitively exhibited similar behavioral performance on no-go trials (i.e., equivalent commission errors) as compared to the younger adults. These results seemed to suggest that the older adults were capable of inhibiting the no-go trials. In addition, based on the no-go commission error data, it appeared to be more difficult for both younger and older adults to withhold a response to cfNOGO stimuli compared to irNOGO stimuli. Yet, since the behavioral no-go performance can only be measured with commission errors, it is interesting to examine whether or not the underlying neural activity for the no-go trials, as reflected by the ERPs, would be also similar, as shown in the behavioral data between the two age groups.

The current ERP data showed that for the younger adults, the cfNOGO stimuli were associated with smaller N2 amplitudes than those of the irNOGO stimuli, specifically for the 20% no-go probability condition. Previous research has argued that the N2 enhancement of the no-go trials either reflects the operation of a cognitive top-down inhibition mechanism needed to suppress the incorrect tendency to respond or reflects an electrophysiological correlate of conflict between go and no-go response representations that is detected in ACC (Nieuwenhuis et al., 2003). Since the current results showed that the cfNOGO trials were associated with smaller N2 amplitudes and were accompanied with more commission errors than the irNOGO trials (but only for the 20% no-go trial condition), this suggests that either the younger adults experienced less conflict and hence recruited less control processes for the cfNOGO trials than irNOGO trials based on the conflict hypothesis of N2 (Falkenstein et al., 2001; Van Veen and Carter, 2002a,b) or they were less able to inhibit the cfNOGO stimuli based on the inhibition account (Roche et al., 2005). Nevertheless, since we additionally observed that N2 peak-to-peak amplitudes were also modulated by the no-go stimulus probability for the younger adults, the results appeared to be more consistent with the results reported by Nieuwenhuis et al. (2003); see also Smith et al. (2008), who suggested that N2 may reflect response conflict rather than inhibition* per se*.

**Figure 9:**
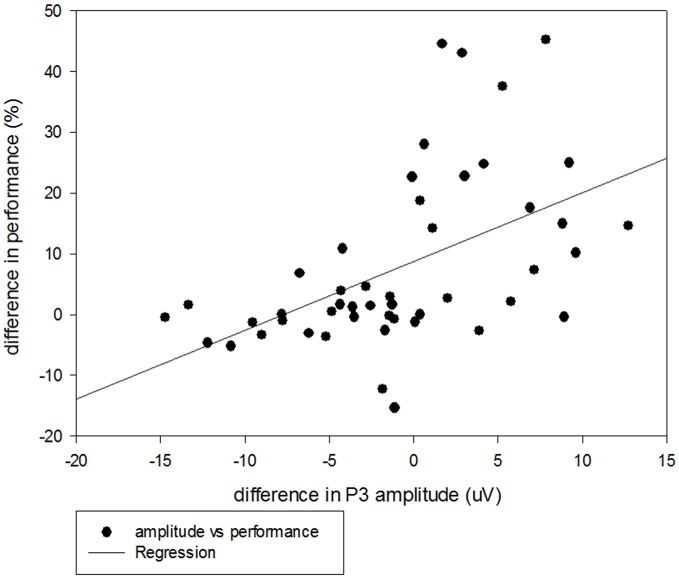
**Scatterplot with regression line illustrating the correlation between P3 amplitudes (uV) and behavioral performance (%) for the irNOGO trials relative to GO trials across all three no-go probability conditions for the older adults**.

More importantly, in the current study, the older adults paradoxically exhibited larger P3 amplitudes for the irNOGO than cfNOGO trials, whereas behaviorally they committed more errors in the cfNOGO than irNOGO trials. This seems to suggest that the older adults recruited more control processes in order to conquer the commitment of responses for the no-go trials, especially for the irNOGO trials. The more direct evidence that supports this speculation comes from the correlational analysis between the behavioral performance and P3 amplitudes. The correlation was significant (*r* = 0.51, *p* < 0.001; see Figure 9) and showed that better performance (i.e., lower PE) inhibiting the no-go trials was associated with larger the P3 amplitudes in the no-go trials, relative to go trials. This finding was consistent with Vallesi et al. (2009), who showed that the irNOGO trials seemed to be more distracting (and hence cause a larger conflict N2) for the older adults to withhold the response; hence, the older adults needed to recruit more control processes in withholding the response towards the irNOGO trials than the cfNOGO trials, as reflected by their larger no-go P3 peak amplitudes for the irNOGO than cfNOGO trials. However, no such phenomenon was found for the younger adults. This age-related compensatory response (i.e., conquering the irNOGO stimuli with the price of poorer performance of the cfNOGO stimuli) were specifically seen in the 20% no-go trial probability condition. The manipulation of the low probability of no-go trials has proved successful in activating frontal lobe structures associated with executive control in other response inhibition tasks (MacDonald et al., 2000). Hence, in this scenario, the older adults might thus recruit more compensatory responses in withholding the responses towards the no-go trials. This might also explain why there was no age effect seen in the behavioral no-go commission errors due to the compensatory responses.

The current finding that the older adults were more distracted by the irNOGO stimuli than the cfNOGO stimuli appears to be consistent with previous reports in the memory research domain, which suggests that older adults (more pronounced after age 65) suffer from increasing vulnerability to internal distraction and weakening concentration skills due to subtle changes in brain activity (Grady et al., 2006). Grady et al. (2006) found that older adults have difficulty activating brain regions necessary for concentration (e.g., reading) and de-activating or tuning down other regions that are associated with internal thoughts (e.g., thinking about yourself, what you did yesterday). Two key regions of the brain that allow the mind to focus on a single task and tune out unwanted thoughts get out of kilter much earlier in life than previously suspected. When the mind pays strict attention, special neural circuits in the prefrontal cortex become more active, and at the same time, related brain areas in the medial frontal lobe (monitoring more general background activity) slack off. Conversely, when the mind is at rest, the level of brain activity in these two regions is then reversed. Such a seesaw activity pattern can begin to break down during memory tasks starting at about age 40.

Another interesting finding of this study is that older adults seemed to devote more effort towards no-go trials, since the no-go probability effect was only seen significantly for the no-go trials, whereas the effect of no-go probability was significant for both the go and no-go trials for the younger adults. While the younger adults exhibited the conventional larger P3 amplitudes on go trials in the low go-prepotency condition than in the high go-prepotency condition, the older adults did not show such a pattern. It is therefore not surprising to observe a significant age effect on the go P3 amplitudes in the 50 and 80% no-go probability conditions rather than in the 20% no-go probability condition (*p* = 0.24). On the other hand, the amplitudes of both N2 and P3 on the no-go trials were found to vary significantly as a function of the relative frequency of no-go probability (20, 50 and 80%) conditions for both age groups. These results suggest that the older adults (similarly to the younger adults) when withholding the responses could be influenced by the no-go trials’ probability. Given that the older adults were not influenced by the go trials’ probability when executing the responses for the go trials but that they were influenced by the no-go trials’ probability when withholding the responses on no-go trials, we could suspect that the older adults seemed to devote more effort to the no-go trials since it was more effortful for them to withhold the responses; hence, they were more sensitive to the no-go trials’ probability.

Some final issues should be noted. First, this study used a between-subjects design for the factor of no-go probability which might underestimate the effect of no-go probability on the current results, and therefore the current interpretations regarding the interactions with the no-go probability should be treated with caution. Second, although the MMSE scores for the elderly participants in the current study were all within the normal range (on average 26–28), one may be still concerned that these elderly might exhibit an early sign of mild cognitive impairment. Nevertheless, since we have run ANCOVAs using MMSE as a covariate factor and the result patterns remained the same as reported here, we believe that although the older adults exhibited lower MMSE scores, they did not confound the current findings. Third, the topography of the no-go P3 in the older adults (Figure 4B) seems to be more frontally distributed compared with the younger adults who showed a more central distribution (Figure 3B). This might be a hint toward a posterior-anterior shift in aging (PASA, see Davis et al., 2008). In order to examine if the current findings were consistent with the theory of PASA, we re-ran ANOVAs for the P3 data with an additional factor of electrode sites of FCz, Fz, and Cz. The results of the 4-way ANOVAs showed no significant interactions of electrode sites with all other factors, except its own significant effect; hence, the current data did not seem to show a PASA effect for the elderly. Finally, it has been reported in previous studies that older adults showed similar (Falkenstein et al., 2002) and sometimes stronger no-go P3 amplitudes (Hong et al., 2014) compared with younger adults. However, in this study, it seems that older adults showed smaller no-go P3 amplitudes (Figure 6B). Yet, it is important to note that Falkenstein et al. (2002) and Hong et al. (2014) used the P3 difference wave (P3d: no-go minus go ERPs) rather than the absolute no-go P3 amplitude, as reported in this study. When we subtracted go ERPs from no-go ERPs, we likewise observed similar P3d amplitudes (in 20% condition) and larger P3d amplitudes (in both 50 and 80% conditions) for the older adults compared with the younger adults. We decided to report go and no-go ERPs separately because of the following reasons: (1) go and no-go trials involve different processes with respect to motor execution and (2) we were more interested in contrasting irNOGO and cfNOGO trials.

To conclude, using ERP data the current study revealed that older adults were more prone to distraction induced by irNOGO stimuli, and therefore they exerted more control processes to conquer such distraction. Furthermore, older adults tended to devote more effort to withholding responses towards the no-go trials, especially in the condition where more control demand was needed (e.g., in the 20% no-go trials’ probability in this study). This study provided a deeper understanding into how older adults adopted strategies in performing the go/no-go task (e.g., devoting attention to no-go trials in order to cope with their deficient inhibition ability). This interpretation appears to be contradictory to the well-known aging hypothesis of inhibition deficits (Hasher and Zacks, 1988). Yet, given that previous research has already demonstrated that older adults would develop a strategy to cope with some task scenarios, we suggest in the current study that older adults performing the go/no-go task adopted a compensating strategy of paying more attention to the no-go trials.

## Ethics Statement

The study was approved by the Ethical committee of the National Cheng Kung University in accordance with the Declaration of Helsinki. Written informed consent was obtained from all subjects before the experiment.

## Author Contributions

SH designed the experiment, wrote the grant proposal, guided in executing the experiment, interpreted the data, and wrote the manuscript; MU analyzed the data, plotted the figures; C-HT conducted the experiment and collected the data.

## Conflict of Interest Statement

The authors declare that the research was conducted in the absence of any commercial or financial relationships that could be construed as a potential conflict of interest.
